# Body composition, lifestyle, and depression: a prospective study in the UK biobank

**DOI:** 10.1186/s12889-024-17891-6

**Published:** 2024-02-06

**Authors:** Xingyu Lv, Jie Cai, Xiang Li, Xuan Wang, Hao Ma, Yoriko Heianza, Lu Qi, Tao Zhou

**Affiliations:** 1 Department of Epidemiology, School of Public Health (Shenzhen), Shenzhen Campus of Sun Yat-sen University, No.66 Gongchang Road, Guangming District, Shenzhen, People’s Republic of China 518107; 2 Department of Epidemiology, School of Public Health and Tropical Medicine, Tulane University, 1440 Canal Street, Suite 1724, New Orleans, LA 70112 USA; 3 Department of Nutrition, Harvard T.H. Chan School of Public Health, Boston, MA USA

**Keywords:** Fat distribution, Mental health, Metabolism, Epidemiology

## Abstract

**Background:**

Obesity has been related to depression and adhering healthy lifestyle was beneficial to lower the risk of depression; however, little is known about the relationship between body composition and fat distribution with depression risk and the influence of body composition and fat distribution on the association of lifestyle and depression. Therefore, we aimed to investigate whether body composition and fat distribution were associated with the adverse events of depression and the relationship between lifestyle and depression.

**Methods:**

We included 330,131 participants without depression at baseline in the UK Biobank (mean age, 56.9 years; 53.83% females). The assessment of depression was sourced from health outcomes across self-report, primary care, hospital inpatient data, and death data. Body composition was determined by bioelectrical impedance. Seven lifestyles (no current smoking, moderate alcohol consumption, regular physical activity, healthy diet, less sedentary behavior, healthy sleep pattern, and appropriate social connection) were used to generate a lifestyle score.

**Results:**

During a median of 11.7 years of follow-up, 7576 incident depression occurred. All the body composition measures were positively associated with depression risk, with the Hazard ratios (HR) for the uppermost tertile (T3) versus the lowest tertile (T1) ranging from 1.26 (95% CI: 1.15–1.39) for trunk fat-free mass (TFFM) to 1.78 (1.62–1.97) for leg fat percentage (LFP). In addition, we found significant interactions between fat mass-related indices, especially leg fat mass (LFM) (*p* = 1.65 × 10^−9^), and lifestyle score on the risk of depression, for which the beneficial associations of a healthy lifestyle with the risk of depression were more evident among participants with low body fat measurement.

**Conclusions:**

High levels of body composition measures were associated with an increased depression risk. Adverse body composition measures may weaken the link between a healthy lifestyle and a reduced risk of depression.

**Supplementary Information:**

The online version contains supplementary material available at 10.1186/s12889-024-17891-6.

## Introduction

Depression has a severe impact not only on the individual level but also on public health and the economy. According to data released by the Health Measurement and Evaluation Study in 2021, approximately 280 million people worldwide suffer from depression, with an estimated 3.8% of the population affected [1]. Depression is also the main cause of suicide deaths, with nearly 0.8 million people committing suicide each year [2].

Previous studies have consistently linked obesity with depression in adult populations [3, 4]. Compared with the general population, adults with obesity are 55% more likely to suffer from depression [5]. A Mendelian randomization study in UK Biobank showed that obesity was a causal risk factor for depression [6]. However, previous studies used body mass index (BMI) as a surrogate for adiposity, under the assumption that body composition is sufficiently similar between individuals. Large inter-individual variability exists in body composition and fat distribution for those with a similar BMI [7]. It has been shown that the difference in body fat mass was more significant than the difference in BMI, suggesting that the latter may underestimate the extent of obesity in this population [8]. A study in a weight loss trial showed that increases in BMI and body fat attenuated the improvement in depression during weight loss [9]. A case-control study indicated that BMI, body fat percentage (BFP), fat mass, fat-free mass, and waist-hip ratio (WHR) were higher in patients with lifetime depression compared to healthy controls [10].

Lifestyle is a common and crucial modifiable risk factor for many diseases. Many researchers applied scores to evaluate the lifestyle and suggested that adhering healthy lifestyle was beneficial for lowering the risk of adverse events [11–13]. Behavioral intervention is a widely accepted treatment for depression [14]. A systematic large-scale research identified a set of modifiable factors associated with depression across social, sleep, dietary, and exercise-related domains, and these factors were supported by Mendelian randomization evidence [15]. However, the selection of limited lifestyle factors [16] in previous studies leaves emerging important factors such as sleep pattern, sedentary behavior, and social connection out of the lifestyle scores in relation to the risk of depression. We sought to apply a new comprehensive lifestyle score, defined following the prevailing guidelines on depression [17], to evaluate the joint impact of lifestyle and body composition on the risk of depression [11].

In this study, we investigated the associations of body composition and fat distribution with depression in the British population. We further examined whether a healthy lifestyle pattern based on seven modifiable lifestyle factors: smoking, diet, alcohol consumption, physical activity, sleep pattern, social connection, and sedentary behavior, could modify the association of body composition with depression.

## Methods

### Study design and population

Our study was conducted among participants from the UK Biobank, a prospective population-based cohort study. The UK Biobank study started in 2006 and, until 2010, recruited more than 500,000 participants aged 37 to 73 years from the general population at 22 assessment centers throughout the United Kingdom. Participants provided information on lifestyle and other potentially health-related aspects through extensive baseline touchscreen and nurse-led questionnaires, physical measurements, and biological samples [18]. In the current study, we included all participants who were classified as British and without a history of reported depression at baseline, and participants without data on genetic and lifestyle factors (smoking, diet, alcohol consumption,  physical activity, sleep pattern, social connection, and sedentary behavior) were excluded.

### Body composition

We examined continuous physiological measures obtained at the baseline assessment, including body mass index (BMI), waist circumference (WC), hip circumference (HC), body fat percentage (BFP), whole-body fat mass (WBFM), whole-body fat-free mass (WBFFM), whole-body water mass (WBWM), trunk fat percentage (TFP), trunk fat mass (TFM), trunk fat-free mass (TFFM), leg fat percentage (LFP), leg fat mass (LFM), leg fat-free mass (LFFM), arm fat percentage (AFP), arm fat mass (AFM), and arm fat-free mass (AFFM). BMI was calculated as weight in kilograms divided by the square of height in meters and classified into three categories based on the World Health Organization’s criteria: underweight/normal (< 25 kg/m^2^), overweight (25 -30 kg/m^2^), and obese (≥ 30 kg/m^2^) [19]. WHR was calculated as waist circumference in centimeters divided by hip circumference in centimeters and classified into three different body shapes: normal weight, peripheral obesity (WHR < 0.76 for females and WHR < 0.87 for males), and abdominal obesity (WHR > 0.84 for females and WHR > 0.99 for males) [20]. Fat mass to fat-free mass ratio (FFR) was calculated as whole body fat mass divided by whole body fat-free mass.

### Lifestyle factors

Our study considered seven modifiable behavioral factors, including four traditional factors (smoking, physical activity, alcohol consumption, and diet) and three novel factors (sleep pattern, sedentary behavior, and social connection [21]) according to previous evidence, to generate a lifestyle score [11, 22]. Details of the assessment of each lifestyle factor can be found in Supplementary Table 1–3.

The standard of low-risk lifestyle in our study included no current smoking, moderate alcohol consumption, regular physical activity, healthy diet, less sedentary behavior, healthy sleep pattern, and appropriate social connection. Regular physical activity was defined as at least 150 min/week of moderate activity or 75 min/week of vigorous activity (or an equivalent combination). Participants who reported no drinking or drinking on special occasions only, and no more than one drink/day for women and two drinks/day for men (one drink is measured as 8 g ethanol in the UK [23]) were defined as moderate drinking. A low-risk level diet was defined as an adequate intake of at least one-half of 10 food groups recommended as follows: increased consumption of fruits, vegetables, whole grains, fish, dairy, and vegetable oils and reduced consumption of refined grains, processed/unprocessed meats, and sugar-sweetened beverages [13, 24]. Healthy sleep patterns were defined by five individual sleep behaviors (sleep duration, chronotype, insomnia, snoring, and excessive daytime sleepiness) [22]. For each sleep behavior, low risk or high risk was coded as 1 or 0, respectively. A healthy sleep score was the sum of the five scores, ranging from 0 to 5. Low-risk sleep behaviors were defined as healthy sleep scores of 4 or 5. We defined a low-risk level of sedentary behavior as the time of television watching < 4 h/day according to a previous study [25], The social connection level was evaluated by the information on the number in the household, frequency of friend/family visits, and participation in leisure/social activity. For each social behavior, low risk or high risk was assigned 1 point and otherwise 0 points, respectively. The three aspects were summed to obtain a social score ranging from 0 to 3, and a score of 2–3 was defined as appropriate social connection [21].

For each factor, a low-risk level was assigned 1 point and otherwise 0 points. The lifestyle score was constructed as the sum of all seven factors, ranging from 0 to 7, with a higher score indicating a healthier lifestyle. Then the lifestyle score was subsequently categorized into unfavorable (0–2), intermediate (3–4), and favorable lifestyles (5–7).

Additionally, to determine the best combination of lifestyle scores, we included components that were significantly associated with the risk of depression to calculate a modified healthy lifestyle score. The score was weighted based on the β coefficient of each lifestyle factor in the Cox proportional hazard model with all seven lifestyle factors, as well as adjustments for other covariance. We applied the Cox proportional hazard regression model to explore the associations of body composition and modified lifestyle score with incident depression, using the modified lifestyle (range from 0 to 5) that included no current smoking, regular physical activity, healthy sleep pattern, appropriate social connection, and less sedentary behavior.

### Assessment of depression

The primary outcome was the incidence of depression during follow-up. The incident depression was sourced from health outcomes across self-report, primary care, hospital inpatient data, and death data, and was identified using ICD code F32.

### Polygenetic risk score

We calculated the polygenetic risk score (PRS) to assess the cumulative genetic risk for depression. The score was constructed based on the Genome-Wide Association Studies (GWAS) summary-statistic data on depression in the Genetic Epidemiology Research on Adult Health and Aging (GERA) [26]. The present study was restricted to British samples in the UK Biobank. We inferred the posterior effect sizes of Single nucleotide polymorphisms (SNPs) with Ldpred2, an approach that quantifies the contribution of each by examining the relationship between test statistics and linkage disequilibrium (LD) [27]. All the included SNPs were in Hardy-Weinberg equilibrium (HWE) with a *p*_*hwe*_ > 1.0 × 10^−6^ and had a minor allele frequency (MAF) ≥ 1%. The PRS for all individuals in UK Biobank was z-standardized.

### Statistical analysis

Continuous variables were reported as means and standard deviations (SDs), while categorical variables as numbers and percentages. We used Cox proportional hazard regression models to determine the associations of body composition, lifestyle, and joint analysis of body composition and lifestyle with the risk of depression. Participants with depression at baseline were excluded. The model was adjusted for age, sex, assessment center, and Townsend Deprivation Index. We tested interactions of the body composition indices and genetic risk score of depression on the risk of depression by including the respective interaction terms in the models, adjusted for age, sex, assessment center, Townsend Deprivation Index, genotyping batch, and the first 10 principal components of ancestry. The proportional hazard assumption was checked by tests based on Schoenfeld residuals, and the results suggested that the assumptions had not been violated.

We undertook a series of sensitivity analyses to evaluate the robustness of our findings. First, the risk of depression was examined by using a modified lifestyle score that excludes variables not significantly associated with the risk of depression (Supplementary Table 4–5). Second, we analyzed the association of body composition with the risk of depression within the sex subgroups. Third, to minimize the possibility of spurious associations due to reverse causation, the associations of body composition, lifestyle, and genetic risk with depression were re-analyzed after excluding the people who were diagnosed during the first two years of follow-up. Finally, we further excluded the participants with depression symptoms at baseline which was assessed by the validated two-item Patient Health Questionnaire (PHQ-2) as a sensitivity analysis. To maximize the likelihood of reporting true findings, we used Benjamini & Hochberg correction to adjust for multiple tests. All analyses were performed using R statistical software (version 4.1.1). The survival [28] and survminer packages were used for Cox proportional hazard regression. The Hmisc [29] and corrplot [30] packages were used for correlation analyses.

## Result

### Population characteristics

At baseline, 502,507 participants were assessed. After excluding non-British participants (*n* = 165,088) without body composition measurements or genetic information (*n* = 7288), a total of 330,131 participants were included in our study. The baseline characteristics of the participants according to incident depression was shown in Table 1. There were 26,902 participants with depression at baseline. The mean (SD) age was 56.87 (7.98) years, and 177,724 (53.83%) were females. In total, 45,440 (13.76%) individuals had a favorable lifestyle, 215,668 (65.33%) had an overall intermediate lifestyle, and 69,023 (20.91%) had an unfavorable lifestyle. A total of 7576 incident cases of depression were documented during a median follow-up time of 11.7 years. Participants who developed depression were more likely to have a higher BMI, BFP, and body and trunk fat mass, while with lower body and trunk fat-free mass, and WBWM at baseline.

**Table 1:** Baseline characteristics of UK Biobank Participant

Baseline Characteristics	All participants (*N* = 330,131)	Incident depression	
		Yes (*N* = 7576)	No (*N* = 295,653)
Age, years	56.87 ± 7.98	55.95 ± 8.23	56.87 ± 7.98
Sex, female, %	177,724 (53.83%)	4636 (61.19%)	155,467 (52.58%)
Townsend deprivation index	−1.58 ± 2.92	−0.89 ± 3.24	−1.6 ± 2.92
Body mass index, kg/m^2^	27.40 ± 4.72	28.34 ± 5.41	27.39 ± 4.73
Body fat percentage, %	31.38 ± 8.49	33.29 ± 8.81	31.35 ± 8.49
Whole-Body fat mass, kg	24.84 ± 9.47	26.98 ± 10.87	24.82 ± 9.49
Whole-Body fat-free mass, kg	53.49 ± 11.53	56.29 ± 11.48	53.49 ± 11.54
Whole-Body water mass, kg	39.14 ± 8.43	38.56 ± 8.39	39.14 ± 8.44
Waist circumstance, cm	90.31 ± 13.40	91.98 ± 14.27	90.28 ± 13.43
Hip circumstance, cm	103.43 ± 9.06	104.81 ± 10.46	103.42 ± 9.07
Trunk fat percentage, %	31.17 ± 7.91	32.72 ± 8.28	31.15 ± 7.92
Trunk fat mass, kg	13.79 ± 5.12	14.66 ± 5.68	13.78 ± 5.13
Trunk fat-free mass, kg	29.74 ± 6.00	29.21 ± 5.88	29.75 ± 6.01
Leg fat percentage, %	31.88 ± 10.59	34.18 ± 10.67	31.53 ± 10.54
Leg fat mass, kg	4.26 ± 1.86	4.74 ± 2.11	4.20 ± 1.81
Leg fat-free mass, kg	8.95 ± 2.02	8.88 ± 2.06	8.98 ± 2.02
Arm fat percentage, %	29.81 ± 10.09	32.30 ± 10.86	29.79 ± 10.09
Arm fat mass, kg	1.28 ± 0.66	1.44 ± 0.82	1.27 ± 0.66
Arm fat-free mass, kg	2.93 ± 0.83	2.87 ± 0.83	2.93 ± 0.83
Lifestyle behaviors, %			
Unfavorable lifestyle	69,023 (20.91%)	2396 (31.63%)	58,367 (19.74%)
Intermediate lifestyle	215,668 (65.33%)	4510 (59.53%)	194,849 (65.90%)
Favorable lifestyle	45,440 (13.76%)	670 (8.84%)	42,437 (14.35%)

* Values are means ± SD unless otherwise indicated

* The lifestyle behaviors were defined according to lifestyle score including seven factors, ranging from 0 to 7, with a higher score indicating better adherence to an overall healthy lifestyle. The lifestyle score was subsequently categorized into unfavorable (0–2), intermediate (3–4), and favorable lifestyles (5–7)

We analyzed the correlations between different body composition indices. The correlations between BMI and WC, HC, and WBFM were higher than 0.8. Both WC and WHR were weakly correlated with whole-body or regional fat mass. We also observed a high correlation coefficient between fat-free mass and WBWM (Supplementary Fig. 1).

### Associations of body composition indices with incident depression

The associations between body composition indices and depression were shown in Supplementary Table 6. We observed that all indices were associated with an increased risk of depression adjusted for age, sex, UK Biobank assessment center, and Townsend Deprivation Index. A 1-SD (4.72 kg/m^2^) higher BMI was associated with a 1.19 (95% CI: 1.02–1.37) higher risk of depression. A 1-SD (8.49) higher BFP was associated with a 1.26 (95% CI: 1.03–1.50) higher risk of depression. The association of a 1-SD increase (10.59) in leg fat percentage with the risk of depression was the most pronounced (HR = 1.43, 95% CI: 1.02–1.79).

**Fig. 1:**
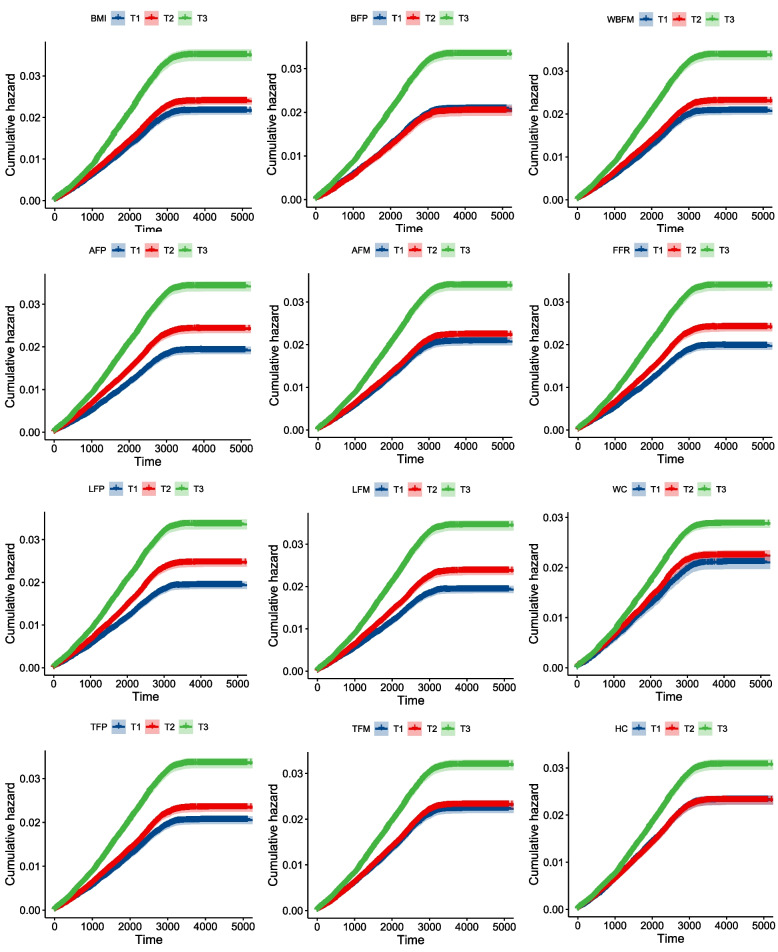
Associations of body composition with the risk of depression. BMI, body mass index; BFP, body fat percentage; WBFM, wholebody fat mass; AFP, arm fat percentage; AFM, arm fat mass; LFP, leg fat percentage; LFM, leg fat mass; TFP, trunk fat percentage; TFM, trunk fat mass; WC, waist circumference; HC, hip circumference; FFR, Fat mass to fat-free mass ratio. Cumulative hazards of depression were estimated from Cox proportional hazards models, adjusted for sex, age, assessment center, and Townsend deprivation index. T1, T2, and T3 correspond to the corresponding tertiles from 0 to 3

Although the results showed that the higher risk of depression was associated with higher body fat indices, the relationship between different body components and depression risk slightly varied. We found that among all the indices, compared with the lowest tertile, the highest tertile of body composition corresponded to the highest level of depression risk (Fig. 1).

### Associations of lifestyle with incident depression

We also explored the associations of lifestyle with the risk of depression (Supplementary Table 5). The results showed that a higher lifestyle score was associated with a reduced risk of depression (HR = 0.76, 95% CI: 0.74–0.77). Except for the diet score, other higher levels of the components of the lifestyle score were significantly associated with a lower risk of depression. Additionally, a healthy sleep pattern was also associated with a lower risk of depression (HR = 0.61, 95% CI: 0.58–0.65).

### Interaction between lifestyle and body composition on the risk of depression

**Fig. 2:**
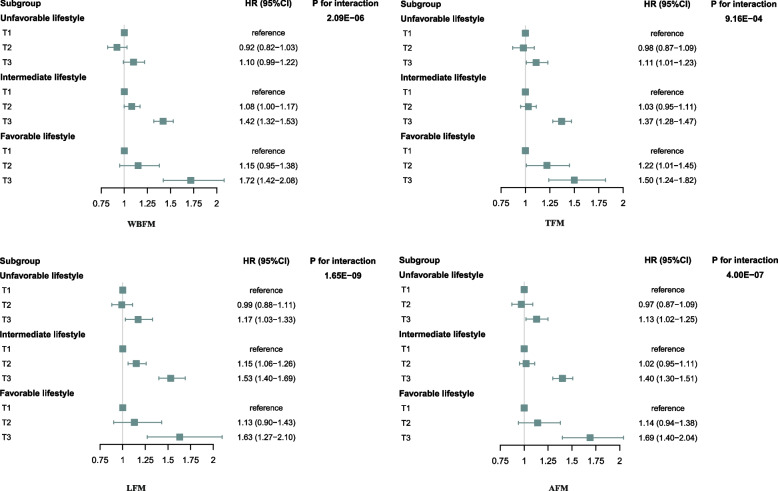
Associations of lifestyle with risks for depression according to body composition and fat distribution. WBFM, whole body fat mass; TFM, trunk fat mass; LFM, leg fat mass; AFM, arm fat mass. Multivariate-adjusted hazard ratios (HRs) and 95% CIs of depression were estimated from Cox proportional hazards models, adjusted for sex, age, assessment center, and Townsend deprivation index. T1, T2, and T3 correspond to the corresponding tertiles from 0 to 3

Based on the multiplicative model, we observed significant interactions between the lifestyle score and whole body, regional fat mass on the risk of depression. The *p*-interaction for the whole body, trunk, arm, and leg fat mass were 2.09 × 10^−6^, 9.16 × 10^−4^, 4.00 × 10^−7^, and 1.65 × 10^−9^, respectively (Fig. 2). There was also a significant interaction between lifestyle and FFR (*p*-interaction = 6.81 × 10^−7^). We found no interactions between lifestyle score and the whole body and regional fat-free mass, WBWM, WC, and WHR. Then we performed the analysis of the associations of body composition indices with the risk of incident depression stratified by lifestyle (Supplementary Table 7). The associations of different body component indices with depression risk varied among participants with different lifestyle grades. The associations of body component indices with the risk of incident depression tended to be more pronounced among those with a healthier lifestyle. Moreover, a higher body fat measurement attenuated the beneficial association of a healthy lifestyle with the risk of depression (Fig. 2).

Furthermore, we jointly analyzed the association of different types of body shapes and lifestyles with the risk of depression (Supplementary Fig. 2). We found that the improvement of lifestyle was important for people with all types of body shapes to lower depression risk. The HRs (95% CI) were 0.56 (0.51–0.62), and 0.37 (0.32–0.43) in intermediate and favorable lifestyles compared with unfavorable lifestyles in people with normal weight, which was the most significant among three body shapes.

### Interaction between genetic risk and lifestyle on incident depression

We then assessed the modification effect of body composition on the association of genetic risk of depression with the incidence of depression, with adjustment of age, sex, UK Biobank assessment center, Townsend Deprivation Index, genotyping batch, and the first ten genetic principal components. We did not observe a significant interaction between body composition indices and the genetic risk of depression shown in Supplementary Table 8.

### Sensitivity analysis

With the modified lifestyle score, the association of body composition and modified lifestyle score with incident depression didn’t change materially (Supplementary Table 9), which suggested that there was no need to modify the original one. In sex-subgroups, almost all body composition indices were related to depression risk in women, slightly different from those in men. The associations of different fat distribution with the risk of depression in both populations change consistently with that of the whole population (Supplementary Table 10). When we excluded participants who developed depression events within the first 2 years of follow-up (*n* = 2879), the associations of body composition and lifestyle with depression remained consistent with the main analysis (Supplementary Table 11–13). The result remained unchanged after we further excluded participants with depression symptom assessment by PHQ-2 (*n* = 10,866) (Supplementary Table 14–16).

## Discussion

In this large-scale prospective study, we found that increasing fat mass, fat percentage, and fat-free mass were all associated with an increased risk of depression, with fat mass showing the strongest relation. We found interactions of lifestyle with BMI, fat percentage, and fat mass indices on the risk of depression. The interaction between leg fat mass and lifestyle was the most significant. The beneficial association of a healthy lifestyle with depression was more pronounced among those with a healthy body fat distribution. Viewed differently, the association of body composition with incident depression was particularly marked among participants with a favorable lifestyle.

Although BMI is widely used to approximate overall body fat, it fails to capture other metabolically relevant aspects of adiposity such as fat distribution, leading to considerable disparity in health outcomes between individuals with similar BMI. In particular, differences in body fat distribution, both the location and subtype of adipose tissue used to store excess calories, have been shown different influences on health outcomes [31]. There is also evidence that specific body fat distributions are significantly linked to adverse consequences [32–35]. Therefore, this study explored the relationship between body composition indices and depression in the form of a cohort study. Our study showed that the whole body and regional fat distribution were related to the increased risk of depression, which was consistent with some previous studies [36, 37]. A Mendelian randomization study suggested that both fat mass and height are causal risk factors for depression, while fat-free mass was not. However, the mechanism between body composition distribution and depression remains unclear. A possible explanation is that fat distribution may lead to depression through the HPA (hypothalamic–pituitary–adrenal) axis, which is consisting of stimulating forward and feedback inhibition loops involving the brain, pituitary, and adrenal glands, and regulates glucocorticoid production [38]. That is, individuals with poor fat distribution tend to produce higher levels of glucocorticoid, which stimulates overactivity of the HPA axis and increases the risk of depression.

Reduced risk of depression by adhering to a healthy lifestyle has been well documented [39]. The role of some conventional lifestyles such as diet [40] and physical activity [41] has been studied. To the best of our knowledge, however, only a limited number of studies have examined the associations of combined healthy lifestyle factors with the risk of developing depression. A recent study explored the role of comprehensive lifestyle scores in reducing the risk of depression, but some important risk factors have been ignored [16]. Our present study applied the comprehensive lifestyle evaluation which included three new factors: sleep patterns [22], sedentary behaviors [42], and social connection [23, 43], which were all related to the risk of depression independently. The comprehensive lifestyle evaluation has been related to low risks of all-cause and cause-specific mortality [11].

Our study showed that body fat distribution indices modified the association of lifestyle with the risk of depression. A significant interaction was also found for fat mass-related indices but not for fat-free mass. The possible explanations about the effect of adverse body composition on the association of lifestyle with depression are: (a) The abnormal fat distribution reflects the abnormal metabolic distribution to some extent, which may offset the beneficial effect of lifestyle improvement on reducing the risk of depression. For example, insulin inactivation was associated with food addiction [44], which can affect adherence to a healthy lifestyle, as well as increase the risk of depression. (b) Body composition may be affected by a healthy lifestyle; and mutually, unfavorable body composition may have an adverse effect on some specific components of lifestyle score. Increased fat mass can affect activity and make it difficult to adhere to an active lifestyle [45]. As physical activity declines and weight gains, the psychological impact of weight stigma and discrimination increases, making it easier to choose high-calorie foods, leading to a vicious cycle of further weight gain and depressive mood [46, 47].

From another perspective, participants with adverse body fat distribution indices showed a greater increased risk of depression when adhering to a favorable lifestyle. As for the association of body composition with depression in population across different lifestyles, the possible explanations are: (c) People who adhere to a favorable lifestyle have fewer factors that increase the risk of depression than those who lead an unfavorable lifestyle [15, 16, 25, 48, 49], and the adverse lifestyles could be a risk factor for adverse body composition and may share similar pathways with adverse body composition, such as metabolic dysfunction, that contribute to depression [50]. So, the role of adverse body composition in increasing depression risk is more pronounced in population with favorable lifestyle.

We also found no significant interaction between genetic risk and body composition suggesting that the genetically predetermined risk of depression can’t be modified by body composition indices. The polygenetic score of depression was calculated based on genetic variants that accounted for a small proportion of the variance in depression, which may partly explain the null interaction between the genetic scores and body composition on depression.

Based on the large sample of UK Biobank participants and the use of standardized protocols for data collection, our study first explored the association of body composition with the risk of depression in a prospective design, which was a major strength of our study. However, several limitations of the current study still need to be considered. First, although we adjusted for known potential confounders in our analysis, there may be unmeasured confounders. Second, considering the heterogeneity in genetic, metabolic conditions, and the lifestyle across ethnicities and regions, the participants included in this study were primarily restricted to British to improve internal validity and therefore may not be generalizable to other populations. Third, we adopted the information of lifestyles at baseline. We could not avoid that lifestyle that might change during follow-up, which would affect the exploration of relevance. Fourth, we dichotomized each lifestyle factor and counted the number of low-risk lifestyle factors, ignoring the difference in the magnitude of association between various lifestyle factors and depression. However, we compared the analyses using modified lifestyle scores with the original score, and no significant differences were observed. Fifth, although participants who developed depression within the first 2 years of follow-up were excluded, the risk of reverse causation was still unavoidable.

In conclusion, our study suggested that body fat distribution was related to the risk of depression, and adherence to a healthy lifestyle, especially among those with healthy fat distribution, was important to reduce the risk of depression. It is promising for policymakers to advise individuals to reduce the risk of depression by paying attention to body fat distribution indices in the future.

### Supplementary Information

**Additional file 1: Supplementary Table 1** Diet component definitions in the UK Biobank study. **Supplementary Table 2** Sleep pattern definitions in the UK Biobank study. **Supplementary Table 3** Social connection definitions in the UK Biobank study. **Supplementary Table 4** The associations between the body composition and lifestyle component with the risk of incidengest depression. **Supplementary Table 5** Associations of lifestyle component with incident depression. **Supplementary Table 6** Associations of body composition and incident depression. **Supplementary Table 7** Associations of body composition with incident depression stratified by lifestyle categories. **Supplementary Table 8** Interactions between the body composition and genetic risk of depression with the risk of incident depression. **Supplementary Table 9** Associations of body composition with incident depression stratified by modified lifestyle score. **Supplementary Table 10** Associations of body composition with incident depression stratified by sex. **Supplementary Table 11** Associations of body composition and incident depression after exclusion of the people diagnosed with depression in first two years. **Supplementary Table 12** Associations of lifestyle component and incident depression after exclusion of the people diagnosed within depression in first two years. **Supplementary Table 13** Associations of body composition and lifestyle with incident depression after exclusion of the people diagnosed with depression in first two years. **Supplementary Table 14** Associations of body composition and incident depression after exclusion of the participants with depression symptoms at baseline assessed by PHQ-2. **Supplementary Table 15** Associations of lifestyle component and incident depression after exclusion of the participants with depression symptoms at baseline assessed by PHQ-2. **Supplementary Table 16** Associations of body composition and lifestyle with incident depression after exclusion of the participants with depression symptoms at baseline assessed by PHQ-2. **Supplementary Figure 1** Correlation between body compositions. **Supplementary Figure 2** Associations of lifestyle with risks for depression stratified by WHR.
