# Supplementary material for: Body composition, lifestyle, and depression: a prospective study in the UK biobank
Source: BMC Public Health. 2024 Feb 6;24:393. doi: 10.1186/s12889-024-17891-6 (PMC10848418; doi:10.1186/s12889-024-17891-6)
Supplement: Supplementary file 1 — Additional file 1: Supplementary Table 1 Diet component definitions in the UK Biobank study. Supplementary Table 2 Sleep pattern definitions in the UK Biobank study. Supplementary Table 3 Social connection definitions in the UK Biobank study. Supplementary Table 4 The associations between the body composition and lifestyle component with the risk of incidengest depression. Supplementary Table 5 Associations of lifestyle component with incident depression. Supplementary Table 6 Associations of body composition and incident depression. Supplementary Table 7 Associations of body composition with incident depression stratified by lifestyle categories. Supplementary Table 8 Interactions between the body composition and genetic risk of depression with the risk of incident depression. Supplementary Table 9 Associations of body composition with incident depression stratified by modified lifestyle score. Supplementary Table 10 Associations of body composition with incident depression stratified by sex. Supplementary Table 11 Associations of body composition and incident depression after exclusion of the people diagnosed with depression in first two years. Supplementary Table 12 Associations of lifestyle component and incident depression after exclusion of the people diagnosed within depression in first two years. Supplementary Table 13 Associations of body composition and lifestyle with incident depression after exclusion of the people diagnosed with depression in first two years. Supplementary Table 14 Associations of body composition and incident depression after exclusion of the participants with depression symptoms at baseline assessed by PHQ-2. Supplementary Table 15 Associations of lifestyle component and incident depression after exclusion of the participants with depression symptoms at baseline assessed by PHQ-2. Supplementary Table 16 Associations of body composition and lifestyle with incident depression after exclusion of the participants with depression symptoms [file 12889_2024_17891_MOESM1_ESM.docx]

**Supplemental Tables 1-16 and Supplemental Figures 1-2 for**

**Body composition, lifestyle, and depression: A prospective study in the UK Biobank**

Xingyu Lv^1^, Jie Cai^1^, Xiang Li^2^, Xuan Wang^2^, Hao Ma^2^, Yoriko Heianza^2^, Lu Qi^2,3*^, Tao Zhou^1,2*^

1. Department of Epidemiology, School of Public Health (Shenzhen), Shenzhen Campus of Sun Yat-sen University, Shenzhen, China
2. Department of Epidemiology, School of Public Health and Tropical Medicine, Tulane University, New Orleans, LA
3. Department of Nutrition, Harvard T.H. Chan School of Public Health, Boston, MA

***Corresponding Authors:**

Email: [lqi1@tulane.edu](about:blank) (LQ)

Email: zhout93@mail.sysu.edu.cn (TZ)

**Supplementary** **Table 1** **Diet component definitions in the UK Biobank study**

| **Diet component** | **Intake goal** | **Field IDs** | **Amount per serving** |
| --- | --- | --- | --- |
| Fruit | ≥ 3 servings/day | 1309 (pieces fresh fruit/day)  1319 (pieces dried fruit/day) | 1309 - 1 piece  1319 - 5 pieces |
| Vegetable | ≥ 3 servings/day | 1289 (tablespoons cooked vegetables/day)  1299 (salad/raw vegetables/day) | 3 heaped tablespoons |
| Whole grains | ≥ 3 servings/day | 1438,1448 (wholemeal/wholegrain bread slices/week)  1458,1468 (brain/oat/muesli cereal bowls/week) | 1438/1448 - 1 slice/day  1458/1468 - 1 bowl/day |
| (Shell) fish | ≥ 2 servings/week | 1329 (oily fish/week)  1339 (non-oily fish/week) | Once/week |
| Dairy | ≥ 2 servings/day | 1408 (cheese/week)  1418(milk type) | 1408 - 1 piece/day  1418 - 1 glass/day if consumption of any type of milk |
| Vegetable oils | ≥ 2 servings/day | 1428 (Flora Pro-Active/Benecol spread)  2654 (Flora Pro-Active/Benecol, soft margarine-, olive oil based-, polyunsaturated/sunflower oil based-, other low/reduced fat spread)  1438 (bread slices/week) | 1 serving/day if in combination with  eating at least 2 slices of bread (ID 1438) |
| Refined grains | ≤ 2 servings/day | 1438, 1448 (white, brown, other bread slices/week)  1458, 1468 (biscuit, other cereals/week) | 1438/1448 - 1 slice/day  1458/1468 - 1 bowl/day |
| Processed meals | ≤ 1 servings/week | 1349 (processed meat/week or daily)  3680 (age when last ate meat) | 1349 - 1 piece/day  3680 - 0 pieces/day if indicated having  never eaten meat |
| Unprocessed meals | ≤ 2 servings/week | 1359 (poultry/week or day)  1369 (beef/week or day)  1379 (lamb or mutton/week or day)  1389 (pork/week or day)  3680 (age when last ate meat) | 1359-1389 - once/week  3680 - 0 pieces/day if indicated having  never eaten meat |
| Sugar-sweetened beverages | Don’t drink | 6144 (never consumes drinks containing sugar) | Only 0 servings were possible here. |

**Supplementary** **Table 2 Sleep pattern definitions in the UK Biobank study**

| **Sleep behavior** | **Field IDs** | **Annotation** |
| --- | --- | --- |
| Sleep duration | 1160 (sleep duration) | > 7-8 hours/day |
| Chronotype | 1180 (Morning/evening person) | 1 - definitely a ‘morning’ person  2 - More a ‘morning’ than ‘evening’ person |
| Insomnia | 1200 (Sleeplessness / insomnia) | 1 - never/rarely |
| Snoring | 1210 (Snoring) | 2 - no |
| Excessive daytime sleepiness | 1220 (Daytime dozing/ sleeping) | 0 - never/rarely  1 - Sometimes |

**Supplementary** **Table 3** **Social connection definitions in the UK Biobank study**

| **Social behavior** | **Field IDs** | **Annotation** |
| --- | --- | --- |
| Based on living alone | 709 (Number in household) | 1 - live alone |
| Contact with family or friends | 1031 (Frequency of friend/family visits) | 4 - about 1 time/week  5 - once every few month  6 - Never or almost never  7 - No friends/family outside household |
| Group participation | 6160 (Leisure/social activities) | -7 - None of above |

**Supplementary** **Table 4. The associations between the body composition and lifestyle component with the risk of incident depression**

| **Variable** | **Score** | **Alcohol** | **Diet** | **Physical** |
| --- | --- | --- | --- | --- |
| Body mass index | 1.04 (1.00-1.08) | 0.87 (0.74-1.01) | 1.00 (1.00-1.00) | 1.02 (1.00-1.03) |
| Body fat percentage | 1.05 (1.00-1.09) | 1.03 (1.00-1.05) | 1.01 (1.00-1.03) | 1.03 (1.00-1.06) |
| Waist circumference | 1.01 (1.00-1.02) | 0.83 (0.64-1.02) | 0.94 (0.87-1.00) | 0.99 (0.97-1.00) |
| Hip circumference | 1.04 (1.00-1.07) | 0.89 (0.78-1.01) | 0.98 (0.97-1.00) | 1.01 (1.00-1.02) |
| Whole body |  |  |  |  |
| Whole body fat mass | 1.05 (1.00-1.09) | 0.93 (0.86-1.00) | 1.01 (1.00-1.02) | 1.03 (1.00-1.06) |
| Whole body fat-free mass | 0.99 (0.97–1.00) | 0.80 (0.58-1.02) | 0.92 (0.83-1.00) | 0.96 (0.93-1.00) |
| Whole body water mass | 0.99 (0.97–1.00) | 0.80 (0.58-1.02) | 0.92 (0.83-1.00) | 0.96 (0.92-1.00) |
| Trunk |  |  |  |  |
| Trunk fat percentage | 1.03 (1.00-1.07) | 1.01 (1.00-1.02) | 0.97 (0.93-1.00) | 1.01 (1.00-1.03) |
| Trunk fat mass | 1.03 (1.00-1.07) | 0.94 (0.89-1.00) | 0.97 (0.94-1.00) | 1.01 (1.00-1.03) |
| Trunk fat free mass | 0.98 (0.97–1.00) | 0.81 (0.60-1.02) | 0.92 (0.83-1.00) | 0.96 (0.93-1.00) |
| Leg |  |  |  |  |
| Leg fat percentage | 1.06 (1.00-1.11) | 1.09 (1.00-1.18) | 1.07 (1.00-1.14) | 1.04 (1.00-1.09) |
| Leg fat mass | 1.06 (1.00-1.11) | 0.93 (0.87-1.00) | 1.05 (1.00-1.10) | 1.05 (1.00-1.10) |
| Leg fat-free mass | 0.99 (0.98–1.00) | 0.80 (0.57-1.02) | 0.92 (0.84-1.00) | 0.97 (0.94-1.00) |
| Arm |  |  |  |  |
| Arm fat percentage | 1.05 (1.00-1.10) | 0.96 (0.92-1.00) | 1.04 (1.00-1.07) | 1.04 (1.00-1.08) |
| Arm fat mass | 1.04 (1.00-1.09) | 0.90 (0.80-1.00) | 1.04 (1.00-1.07) | 1.04 (1.00-1.07) |
| Arm fat-free mass | 0.99 (0.97–1.00) | 0.83 (0.65-1.02) | 0.92 (0.83-1.00) | 0.96 (0.92-1.00) |
| FFR | 1.05 (1.00-1.10) | 0.98 (0.96-1.00) | 1.03 (1.00-1.05) | 1.05 (1.00-1.09) |
| WHR | 0.98 (0.96–1.00) | 0.82 (0.63-1.02) | 0.92 (0.84-1.00) | 0.97 (0.94-1.00) |

**Supplementary Table 4 (continue)**

| **Variable** | **Sleep** | **Smoke** | **Social** | **Sedentary** |
| --- | --- | --- | --- | --- |
| Body mass index | 1.06 (1.00-1.12) | 1.17 (1.01-1.32) | 1.08 (1.00-1.15) | 1.05 (1.00-1.11) |
| Body fat percentage | 1.02 (1.00-1.04) | 1.15 (1.01-1.28) | 1.09 (1.00-1.18) | 1.05 (1.00-1.11) |
| Waist circumference | 1.03 (1.00-1.06) | 1.13 (1.01-1.25) | 1.04 (1.00-1.09) | 1.01 (1.00-1.02) |
| Hip circumference | 1.05 (1.00-1.10) | 1.16 (1.01-1.31) | 1.09 (1.00-1.17) | 1.02 (1.00-1.05) |
| Whole body |  |  |  |  |
| Whole body fat mass | 1.04 (1.00-1.08) | 1.17 (1.01-1.33) | 1.09 (1.00-1.17) | 1.05 (1.00-1.10) |
| Whole body fat-free mass | 1.02 (1.00-1.05) | 1.08 (1.00-1.15) | 0.98 (0.96-1.00) | 0.97 (0.94-1.00) |
| Whole body water mass | 1.02 (1.00-1.04) | 1.07 (1.00-1.15) | 0.98 (0.96-1.00) | 0.97 (0.94-1.00) |
| Trunk |  |  |  |  |
| Trunk fat percentage | 1.02 (1.00-1.04) | 1.15 (1.01-1.29) | 1.07 (1.00-1.15) | 1.03 (1.00-1.07) |
| Trunk fat mass | 1.03 (1.00-1.06) | 1.17 (1.01-1.33) | 1.07 (1.00-1.13) | 1.03 (1.00-1.06) |
| Trunk fat free mass | 1.02 (1.00-1.04) | 1.06 (1.00-1.12) | 0.98 (0.96-1.00) | 0.97 (0.94-1.00) |
| Leg |  |  |  |  |
| Leg fat percentage | 1.02 (1.00-1.04) | 1.12 (1.01-1.24) | 1.09 (1.00-1.18) | 1.08 (1.00-1.15) |
| Leg fat mass | 1.05 (1.00-1.10) | 1.14 (1.01-1.28) | 1.09 (1.00-1.18) | 1.07 (1.00-1.14) |
| Leg fat-free mass | 1.03 (1.00-1.06) | 1.09 (1.00-1.17) | 0.99 (0.98-1.00) | 0.98 (0.95-1.00) |
| Arm |  |  |  |  |
| Arm fat percentage | 1.03 (1.00-1.05) | 1.13 (1.01-1.26) | 1.10 (1.01-1.20) | 1.05 (1.00-1.11) |
| Arm fat mass | 1.06 (1.00-1.11) | 1.13 (1.01-1.26) | 1.08 (1.00-1.16) | 1.04 (1.00-1.08) |
| Arm fat-free mass | 1.03 (1.00-1.05) | 1.08 (1.00-1.15) | 0.97 (0.95-1.00) | 0.98 (0.96-1.00) |
| FFR | 1.03 (1.00-1.06) | 1.12 (1.01-1.24) | 1.09 (1.00-1.18) | 1.06 (1.00-1.12) |
| WHR | 1.00 (1.00-1.00) | 1.03 (1.00-1.05) | 0.97 (0.94-1.00) | 1.00 (1.00-1.00) |

* WHR, waist circumstance/hip circumstance; FFR, whole body fat mass/whole fat-free mass; HR, hazard ratio.

* Values are HR (95%CI) obtained from Cox proportional hazard regression model, adjusted for sex, age, assessment center, and Townsend deprivation index.

**Supplementary** **Table 5. Associations of lifestyle component with incident depression**

| **Variable** | **HR (95% CI)** | ***P*** |
| --- | --- | --- |
| Lifestyle score | 0.76 (0.74-0.77) | 6.77E-153 |
| Diet score | 1.01 (0.90-1.14) | 8.77E-01 |
| Sleep score | 0.61 (0.58-0.65) | 5.75E-73 |
| Physical score | 0.82 (0.78-0.85) | 3.20E-18 |
| Sedentary score | 0.71 (0.68-0.75) | 4.16E-43 |
| Social score | 0.74 (0.70-0.79) | 6.88E-22 |
| Smoke score | 0.57 (0.54-0.61) | 3.48E-70 |
| Drink score | 0.83 (0.70-0.99) | 4.08E-02 |

* Values are HR (95%CI) per unit increase obtained from Cox proportional hazard regression model, adjusted for sex, age, ethnicity, assessment center, and Townsend deprivation index. HR, hazard ratio.

**Supplementary** **Table 6. Associations of body composition and incident depression**

| **Variable** | **HR (95%CI)** | ***P*** |
| --- | --- | --- |
| Body mass index | 1.19 (1.02-1.37) | 2.67E-66 |
| Body fat percentage | 1.26 (1.03-1.50) | 6.85E-51 |
| Waist circumference | 1.27 (1.03-1.51) | 1.06E-86 |
| Hip circumference | 1.15 (1.01-1.28) | 1.21E-39 |
| Whole body |  |  |
| Whole body fat mass | 1.19 (1.02-1.37) | 7.28E-63 |
| Whole body fat free mass | 1.19 (1.02-1.37) | 7.95E-17 |
| Whole body water mass | 1.19 (1.02-1.37) | 7.09E-17 |
| Trunk |  |  |
| Trunk fat percentage | 1.19 (1.02-1.36) | 8.48E-41 |
| Trunk fat mass | 1.18 (1.01-1.35) | 1.84E-53 |
| Trunk fat free mass | 1.14 (1.07-1.27) | 1.75E-09 |
| Leg |  |  |
| Leg fat percentage | 1.43 (1.02-1.79) | 2.18E-55 |
| Leg fat mass | 1.22 (1.02-1.42) | 6.63E-67 |
| Leg fat-free mass | 1.22 (1.02-1.41) | 7.38E-26 |
| Arm |  |  |
| Arm fat percentage | 1.27 (1.03-1.51) | 3.77E-58 |
| Arm fat mass | 1.18 (1.01-1.34) | 2.91E-69 |
| Arm fat-free mass | 1.20 (1.02-1.38) | 1.62E-17 |
| FFR | 1.25 (1.03-1.47) | 1.53E-56 |
| WHR | 1.32 (1.04-1.60) | 2.52E-79 |

* WHR, waist circumstance/hip circumstance; FFR, whole body fat mass/whole fat-free mass HR, hazard ratio.* Values are HR (95%CI) per SD increase obtained from Cox proportional hazard regression model, adjusted for sex, age, assessment center, and Townsend deprivation index.


**Supplementary** **Table 7. Associations of body composition with incident depression stratified by lifestyle categories**

| **Variable** | **HR (95%CI)** | ***P*** |
| --- | --- | --- |
| **Body mass index** |  | **9.71E-06** |
| Unfavorable lifestyle | 1.08 (1.00-1.15) | 1.64E-05 |
| Intermediate lifestyle | 1.20 (1.02-1.38) | 3.31E-39 |
| Favorable lifestyle | 1.26 (1.03-1.49) | 2.52E-08 |
| **Body fat percentage** |  | **6.17E-06** |
| Unfavorable lifestyle | 1.10 (1.01-1.20) | 3.06E-04 |
| Intermediate lifestyle | 1.24 (1.03-1.46) | 8.88E-26 |
| Favorable lifestyle | 1.24 (1.03-1.46) | 6.70E-05 |
| **Whole body fat mass** |  | **2.09E-06** |
| Unfavorable lifestyle | 1.08 (1.00-1.15) | 4.20E-05 |
| Intermediate lifestyle | 1.20 (1.02-1.38) | 2.26E-36 |
| Favorable lifestyle | 1.23 (1.02-1.43) | 1.26E-06 |
| **Whole body fat-free mass** |  | **2.14E-01** |
| Unfavorable lifestyle | 1.04 (1.00-1.09) | 2.23E-01 |
| Intermediate lifestyle | 1.25 (1.03-1.47) | 4.46E-15 |
| Favorable lifestyle | 1.29 (1.04-1.55) | 1.65E-03 |
| **Whole body water mass** |  | **1.84E-01** |
| Unfavorable lifestyle | 1.05 (1.00-1.09) | 1.85E-01 |
| Intermediate lifestyle | 1.24 (1.03-1.46) | 5.35E-15 |
| Favorable lifestyle | 1.28 (1.03-1.52) | 2.48E-03 |
| **Trunk fat percentage** |  | **8.67E-04** |
| Unfavorable lifestyle | 1.07 (1.00-1.15) | 1.76E-03 |
| Intermediate lifestyle | 1.17 (1.01-1.33) | 1.88E-20 |
| Favorable lifestyle | 1.16 (1.01-1.31) | 6.73E-04 |
| **Trunk fat mass** |  | **9.16E-04** |
| Unfavorable lifestyle | 1.07 (1.00-1.13) | 3.61E-04 |
| Intermediate lifestyle | 1.18 (1.01-1.35) | 1.57E-30 |
| Favorable lifestyle | 1.20 (1.02-1.38) | 1.60E-05 |
| **Trunk fat-free mass** |  | **1.46E-01** |
| Unfavorable lifestyle | 1.02 (1.00-1.04) | 6.15E-01 |
| Intermediate lifestyle | 1.19 (1.02-1.37) | 5.75E-10 |
| Favorable lifestyle | 1.24 (1.02-1.45) | 8.61E-03 |
| **Leg fat percentage** |  | **1.52E-07** |
| Unfavorable lifestyle | 1.15 (1.01-1.29) | 3.78E-04 |
| Intermediate lifestyle | 1.40 (1.06-1.74) | 2.87E-28 |
| Favorable lifestyle | 1.46 (1.08-1.84) | 4.95E-06 |

| **Supplementary Table 7 (continue)** | | |
| --- | --- | --- |
| **Variable** | **HR (95%CI)** | ***P*** |
| **Leg fat mass** |  | **1.65E-09** |
| Unfavorable lifestyle | 1.09 (1.00-1.18) | 2.07E-05 |
| Intermediate lifestyle | 1.23 (1.02-1.44) | 2.40E-39 |
| Favorable lifestyle | 1.29 (1.04-1.54) | 1.63E-07 |
| **Leg fat-free mass** |  | **4.41E-01** |
| Unfavorable lifestyle | 1.07 (1.00-1.13) | 3.91E-02 |
| Intermediate lifestyle | 1.26 (1.03-1.49) | 1.46E-20 |
| Favorable lifestyle | 1.30 (1.04-1.56) | 3.80E-04 |
| **Arm fat percentage** |  | **4.79E-07** |
| Unfavorable lifestyle | 1.12 (1.01-1.23) | 1.50E-05 |
| Intermediate lifestyle | 1.26 (1.03-1.49) | 1.55E-30 |
| Favorable lifestyle | 1.28 (1.03-1.52) | 5.30E-06 |
| **Arm fat mass** |  | **4.00E-07** |
| Unfavorable lifestyle | 1.08 (1.00-1.16) | 1.67E-06 |
| Intermediate lifestyle | 1.18 (1.01-1.35) | 4.98E-41 |
| Favorable lifestyle | 1.24 (1.02-1.45) | 1.60E-07 |
| **Arm fat-free mass** |  | **2.21E-01** |
| Unfavorable lifestyle | 1.04 (1.00-1.08) | 2.68E-01 |
| Intermediate lifestyle | 1.24 (1.03-1.46) | 1.44E-14 |
| Favorable lifestyle | 1.32 (1.04-1.59) | 7.58E-04 |
| **WHR** |  | **6.52E-02** |
| Unfavorable lifestyle | 1.24 (1.03-1.46) | 2.36E-16 |
| Intermediate lifestyle | 1.26 (1.03-1.50) | 3.59E-32 |
| Favorable lifestyle | 1.32 (1.04-1.59) | 3.12E-07 |
| **FFR** |  | **6.81E-07** |
| Unfavorable lifestyle | 1.10 (1.00-1.20) | 7.19E-05 |
| Intermediate lifestyle | 1.24 (1.02-1.45) | 1.92E-29 |
| Favorable lifestyle | 1.26 (1.03-1.49) | 1.06E-05 |

* WHR, waist circumstance/hip circumstance; FFR, whole body fat mass/whole fat-free mass; HR, hazard ratio.

* Values are HR (95%CI) per SD increase obtained from Cox proportional hazard regression model, adjusted for sex, age, assessment center, and Townsend deprivation index.

**Supplementary** **Table 8. Interactions between the** **body composition and genetic risk of depression with the risk of incident depression**

| **Variable** | ***P*** |
| --- | --- |
| Body mass index | 0.36 |
| Body fat percentage | 0.11 |
| Waist circumference | 0.10 |
| Hip circumference | 0.11 |
| Whole body |  |
| Whole body fat mass | 0.10 |
| Whole body fat free mass | 0.12 |
| Whole body water mass | 0.12 |
| Trunk |  |
| Trunk fat percentage | 0.11 |
| Trunk fat mass | 0.10 |
| Trunk fat free mass | 0.12 |
| Leg |  |
| Leg fat percentage | 0.10 |
| Leg fat mass | 0.10 |
| Leg fat-free mass | 0.12 |
| Arm |  |
| Arm fat percentage | 0.11 |
| Arm fat mass | 0.10 |
| Arm fat-free mass | 0.11 |
| FFR | 0.10 |
| WHR | 0.11 |

* WHR, waist circumstance/hip circumstance; FFR, whole body fat mass/whole fat-free mass; HR, hazard ratio.

* *p* values are obtained from Cox proportional hazard regression model, adjusted for sex, age, assessment center, and Townsend deprivation index.

**Supplementary** **Table 9. Associations of body composition with incident depression stratified by modified lifestyle score**

| **Variable** | **HR (95%CI)** | ***P*** |
| --- | --- | --- |
| **Body mass index** |  | **9.49E-07** |
| Unfavorable lifestyle | 1.08 (1.00-1.15) | 1.89E-03 |
| Intermediate lifestyle | 1.18 (1.01-1.34) | 1.20E-20 |
| Favorable lifestyle | 1.23 (1.02-1.43) | 1.34E-23 |
| **Body fat percentage** |  | **5.43E-06** |
| Unfavorable lifestyle | 1.10 (1.00-1.20) | 2.89E-04 |
| Intermediate lifestyle | 1.23 (1.02-1.43) | 6.05E-14 |
| Favorable lifestyle | 1.24 (1.02-1.45) | 3.45E-14 |
| **Whole body fat mass** |  | **4.16E-07** |
| Unfavorable lifestyle | 1.08 (1.00-1.15) | 2.49E-05 |
| Intermediate lifestyle | 1.18 (1.02-1.34) | 2.41E-19 |
| Favorable lifestyle | 1.21 (1.02-1.41) | 3.40E-20 |
| **Whole body fat-free mass** |  | **4.97E-01** |
| Unfavorable lifestyle | 1.05 (1.00-1.09) | 1.78E-01 |
| Intermediate lifestyle | 1.23 (1.02-1.44) | 5.73E-09 |
| Favorable lifestyle | 1.26 (1.03-1.41) | 1.18E-08 |
| **Whole body water mass** |  | **4.46E-01** |
| Unfavorable lifestyle | 1.05 (1.00-1.10) | 1.45E-01 |
| Intermediate lifestyle | 1.23 (1.02-1.44) | 3.88E-09 |
| Favorable lifestyle | 1.25 (1.03-1.47) | 3.29E-08 |
| **Trunk fat percentage** |  | **3.41E-04** |
| Unfavorable lifestyle | 1.07 (1.00-1.14) | 1.89E-03 |
| Intermediate lifestyle | 1.17 (1.01-1.32) | 4.37E-12 |
| Favorable lifestyle | 1.16 (1.01-1.30) | 2.32E-10 |
| **Trunk fat mass** |  | **1.85E-04** |
| Unfavorable lifestyle | 1.07 (1.00-1.14) | 2.32E-04 |
| Intermediate lifestyle | 1.17 (1.01-1.33) | 2.78E-17 |
| Favorable lifestyle | 1.18 (1.01-1.35) | 8.69E-16 |
| **Trunk fat-free mass** |  | **3.63E-01** |
| Unfavorable lifestyle | 1.02 (1.00-1.05) | 5.20E-01 |
| Intermediate lifestyle | 1.17 (1.01-1.33) | 1.32E-05 |
| Favorable lifestyle | 1.22 (1.02-1.41) | 1.10E-06 |
| **Leg fat percentage** |  | **5.82E-07** |
| Unfavorable lifestyle | 1.15 (1.01-1.29) | 2.80E-04 |
| Intermediate lifestyle | 1.34 (1.05-1.63) | 1.85E-13 |
| Favorable lifestyle | 1.45 (1.08-1.81) | 4.50E-18 |

| **Supplementary** **Table 9 (continue)** | | |
| --- | --- | --- |
| **Variable** | **HR (95%CI)** | ***P*** |
| **Leg fat mass** |  | **7.38E-10** |
| Unfavorable lifestyle | 1.09 (1.00-1.18) | 1.22E-05 |
| Intermediate lifestyle | 1.20 (1.02-1.38) | 2.21E-19 |
| Favorable lifestyle | 1.27 (1.03-1.51) | 5.89E-24 |
| **Leg fat-free mass** |  | **8.51E-01** |
| Unfavorable lifestyle | 1.07 (1.00-1.13) | 2.98E-02 |
| Intermediate lifestyle | 1.25 (1.03-1.47) | 1.25E-12 |
| Favorable lifestyle | 1.26 (1.03-1.50) | 1.43E-10 |
| **Arm fat percentage** |  | **3.08E-07** |
| Unfavorable lifestyle | 1.12 (1.01-1.23) | 1.27E-05 |
| Intermediate lifestyle | 1.23 (1.02-1.44) | 1.18E-15 |
| Favorable lifestyle | 1.27 (1.03-1.51) | 5.08E-18 |
| **Arm fat mass** |  | **7.24E-08** |
| Unfavorable lifestyle | 1.08 (1.00-1.16) | 8.58E-07 |
| Intermediate lifestyle | 1.16 (1.01-1.30) | 2.01E-20 |
| Favorable lifestyle | 1.22 (1.02-1.42) | 3.86E-24 |
| **Arm fat-free mass** |  | **5.00E-01** |
| Unfavorable lifestyle | 1.04 (1.00-1.09) | 2.22E-01 |
| Intermediate lifestyle | 1.24 (1.03-1.46) | 2.11E-09 |
| Favorable lifestyle | 1.24 (1.03-1.46) | 7.29E-08 |
| **WHR** |  | **2.21E-01** |
| Unfavorable lifestyle | 1.24 (1.02-1.45) | 2.11E-16 |
| Intermediate lifestyle | 1.26 (1.03-1.49) | 2.36E-19 |
| Favorable lifestyle | 1.27 (1.03-1.51) | 1.30E-17 |
| **FFR** |  | **4.81E-07** |
| Unfavorable lifestyle | 1.10 (1.00-1.20) | 5.96E-05 |
| Intermediate lifestyle | 1.22 (1.02-1.41) | 1.41E-15 |
| Favorable lifestyle | 1.27 (1.03-1.51) | 1.30E-17 |

* WHR, waist circumstance/hip circumstance; FFR, whole body fat mass/whole fat-free mass; HR, hazard ratio.

* Values are HR (95%CI) per SD increase obtained from Cox proportional hazard regression model, adjusted for sex, age, assessment center, and Townsend deprivation index.

**Supplementary** **Table 10. Associations of body composition with incident depression stratified by sex**

| **Variable** | **Female** | | **Male** | |
| --- | --- | --- | --- | --- |
|  | **HR (95%CI)** | ***P*** | **HR (95%CI)** | ***P*** |
| Body mass index | 1.20 (1.02-1.38) | 4.74E-50 | 2.59 (1.64-3.54) | 1.74E-14 |
| Body fat percentage | 1.27 (1.03-1.51) | 2.92E-36 | 4.75 (3.19-6.30) | 1.21E-13 |
| Waist circumference | 1.28 (1.03-1.53) | 9.66E-62 | 11.88 (9.41-14.36) | 1.34E-10 |
| Hip circumference | 1.16 (1.01-1.30) | 8.31E-34 | 2.18 (1.4-2.96) | 4.51E-04 |
| Whole body |  |  |  |  |
| Whole body fat mass | 1.20 (1.02-1.38) | 2.13E-46 | 4.95 (3.35-6.54) | 3.80E-13 |
| Whole body fat free mass | 1.37 (1.06-1.69) | 7.32E-22 | 1.38 (1.06-1.70) | 4.95E-01 |
| Whole body water mass | 1.38 (1.06-1.69) | 3.36E-22 | 1.28 (1.03-1.53) | 4.71E-01 |
| Trunk |  |  |  |  |
| Trunk fat percentage | 1.19 (1.02-1.36) | 5.23E-28 | 5.71 (3.97-7.46) | 7.00E-18 |
| Trunk fat mass | 1.20 (1.02-1.37) | 2.58E-39 | 2.11 (1.36-2.85) | 5.22E-10 |
| Trunk fat free mass | 1.32 (1.04-1.59) | 3.32E-16 | 1.04 (1.00-1.07) | 8.79E-01 |
| Leg |  |  |  |  |
| Leg fat percentage | 1.49 (1.09-1.90) | 2.14E-42 | 1.35 (1.05-1.65) | 7.47E-16 |
| Leg fat mass | 1.22 (1.02-1.42) | 5.74E-50 | 1.24 (1.02-1.45) | 8.41E-20 |
| Leg fat-free mass | 1.35 (1.05-1.65) | 2.71E-25 | 1.14 (1.01-1.27) | 1.96E-07 |
| Arm |  |  |  |  |
| Arm fat percentage | 1.26 (1.03-1.48) | 2.30E-41 | 1.37 (1.05-1.68) | 9.43E-22 |
| Arm fat mass | 1.17 (1.01-1.33) | 4.99E-50 | 1.20 (1.02-1.39) | 2.14E-23 |
| Arm fat-free mass | 1.46 (1.08-1.84) | 3.76E-25 | 1.10 (1.00-1.19) | 6.87E-04 |
| FFR | 1.24 (1.02-1.45) | 1.61E-41 | 1.34 (1.05-1.64) | 4.05E-19 |
| WHR | 1.30 (1.04-1.57) | 1.56E-45 | 1.36 (1.05-1.67) | 8.64E-38 |

* WHR, waist circumstance/hip circumstance; FFR, whole body fat mass/whole fat-free mass; HR, hazard ratio.

* Values are HR (95%CI) per SD increase obtained from Cox proportional hazard regression model, adjusted for age, assessment center, and Townsend deprivation index.

**Supplementary** **Table 11. Associations of body composition and incident depression after exclusion of the people diagnosed with depression in first two years**

| **Variable** | **HR (95%CI)** | ***P*** |
| --- | --- | --- |
| Body mass index | 1.21 (1.18-1.23) | 5.27E-61 |
| Body fat percentage | 1.28 (1.24-1.31) | 4.41E-45 |
| Waist circumference | 1.29 (1.26-1.31) | 3.41E-79 |
| Hip circumference | 1.16 (1.13-1.18) | 2.90E-36 |
| Whole body |  |  |
| Whole body fat mass | 1.21 (1.18-1.23) | 5.04E-57 |
| Whole body fat free mass | 1.23 (1.18-1.27) | 2.71E-18 |
| Whole body water mass | 1.22 (1.18-1.27) | 3.74E-18 |
| Trunk |  |  |
| Trunk fat percentage | 1.20 (1.17-1.22) | 1.70E-35 |
| Trunk fat mass | 1.19 (1.17-1.21) | 2.73E-48 |
| Trunk fat free mass | 1.17 (1.12-1.22) | 6.51E-11 |
| Leg |  |  |
| Leg fat percentage | 1.47 (1.42-1.52) | 1.69E-52 |
| Leg fat mass | 1.24 (1.21-1.26) | 3.19E-62 |
| Leg fat-free mass | 1.25 (1.21-1.29) | 1.87E-26 |
| Arm |  |  |
| Arm fat percentage | 1.28 (1.25-1.31) | 7.85E-50 |
| Arm fat mass | 1.18 (1.16-1.20) | 1.56E-60 |
| Arm fat-free mass | 1.24 (1.19-1.29) | 8.70E-20 |
| FFR | 1.25 (1.03-1.47) | 1.39E-49 |
| WHR | 1.34 (1.31-1.37) | 1.45E-71 |

* WHR, waist circumstance/hip circumstance; FFR, whole body fat mass/whole fat-free mass; HR, hazard ratio.

* Values are HR (95%CI) per SD increase obtained from Cox proportional hazard regression model, adjusted for sex, age, assessment center, and Townsend deprivation index.

**Supplementary** **Table 12. Associations of lifestyle component and incident depression after exclusion of the people diagnosed within depression in first two years**

| **Variable** | **HR (95% CI)** | ***P*** |
| --- | --- | --- |
| Lifestyle score | 0.76 (0.74-0.78) | 5.53E-122 |
| Diet score | 0.97 (0.85-1.11) | 6.77E-01 |
| Sleep score | 0.62 (0.59-0.66) | 1.41E-55 |
| Physical score | 0.82 (0.78-0.86) | 3.23E-14 |
| Sedentary score | 0.72 (0.69-0.76) | 7.38E-32 |
| Social score | 0.74 (0.69-0.79) | 1.62E-19 |
| Smoke score | 0.56 (0.53-0.60) | 2.39E-61 |
| Drink score | 0.82 (0.67-1.00) | 4.53E-02 |

* Values are HR (95%CI) per unit increase obtained from Cox proportional hazard regression model, adjusted for sex, age, assessment center, and Townsend deprivation index. HR, hazard ratio.

**Supplementary** **Table 13. Associations of body composition and lifestyle with incident depression after exclusion of the people diagnosed with depression in first two years**

| **Variable** | **HR (95%CI)** | ***P*** |
| --- | --- | --- |
| **Body mass index** |  | **5.78E-05** |
| Unfavorable lifestyle | 1.09 (1.05-1.13) | 8.83E-06 |
| Intermediate lifestyle | 1.21 (1.18-1.24) | 7.51E-37 |
| Favorable lifestyle | 1.26 (1.17-1.35) | 4.42E-07 |
| **Body fat percentage** |  | **7.70E-05** |
| Unfavorable lifestyle | 1.12 (1.06-1.18) | 2.25E-04 |
| Intermediate lifestyle | 1.26 (1.21-1.30) | 2.02E-23 |
| Favorable lifestyle | 1.24 (1.12-1.36) | 4.36E-04 |
| **Whole body fat mass** |  | **4.09E-05** |
| Unfavorable lifestyle | 1.09 (1.05-1.13) | 1.77E-05 |
| Intermediate lifestyle | 1.21 (1.18-1.24) | 1.03E-33 |
| Favorable lifestyle | 1.22 (1.13-1.31) | 3.09E-05 |
| **Whole body fat-free mass** |  | **1.75E-01** |
| Unfavorable lifestyle | 1.08 (1.01-1.16) | 3.89E-02 |
| Intermediate lifestyle | 1.28 (1.22-1.34) | 8.22E-16 |
| Favorable lifestyle | 1.24 (1.06-1.41) | 2.04E-02 |
| **Whole body water mass** |  | **1.50E-01** |
| Unfavorable lifestyle | 1.09 (1.01-1.16) | 3.14E-02 |
| Intermediate lifestyle | 1.28 (1.22-1.34) | 1.57E-15 |
| Favorable lifestyle | 1.22 (1.05-1.40) | 2.64E-02 |
| **Trunk fat percentage** |  | **3.26E-03** |
| Unfavorable lifestyle | 1.18 (1.14-1.22) | 1.99E-18 |
| Intermediate lifestyle | 1.17 (1.01-1.33) | 1.88E-20 |
| Favorable lifestyle | 1.16 (1.06-1.25) | 3.38E-03 |
| **Trunk fat mass** |  | **5.09E-03** |
| Unfavorable lifestyle | 1.19 (1.16-1.22) | 2.37E-28 |
| Intermediate lifestyle | 1.18 (1.01-1.35) | 1.57E-30 |
| Favorable lifestyle | 1.18 (1.09-1.28) | 3.34E-04 |
| **Trunk fat-free mass** |  | **9.99E-02** |
| Unfavorable lifestyle | 1.06 (0.98-1.14) | 1.32E-01 |
| Intermediate lifestyle | 1.22 (1.16-1.29) | 1.55E-10 |
| Favorable lifestyle | 1.17 (1.00-1.35) | 7.80E-02 |
| **Leg fat percentage** |  | **5.79E-06** |
| Unfavorable lifestyle | 1.20 (1.11-1.29) | 4.21E-05 |
| Intermediate lifestyle | 1.44 (1.38-1.51) | 3.47E-27 |
| Favorable lifestyle | 1.46 (1.28-1.64) | 4.78E-05 |

| **Supplementary Table 13 (continue)** | | |
| --- | --- | --- |
| **Variable** | **HR (95%CI)** | ***P*** |
| **Leg fat mass** |  | **1.25E-07** |
| Unfavorable lifestyle | 1.11 (1.06-1.15) | 5.21E-06 |
| Intermediate lifestyle | 1.25 (1.21-1.28) | 3.42E-37 |
| Favorable lifestyle | 1.29 (1.18-1.39) | 3.70E-06 |
| **Leg fat-free mass** |  | **4.36E-01** |
| Unfavorable lifestyle | 1.09 (1.02-1.16) | 1.03E-02 |
| Intermediate lifestyle | 1.29 (1.24-1.35) | 3.54E-21 |
| Favorable lifestyle | 1.27 (1.11-1.44) | 3.96E-03 |
| **Arm fat percentage** |  | **9.37E-06** |
| Unfavorable lifestyle | 1.13 (1.07-1.19) | 2.90E-05 |
| Intermediate lifestyle | 1.26 (1.22-1.31) | 2.21E-26 |
| Favorable lifestyle | 1.28 (1.17-1.40) | 2.66E-05 |
| **Arm fat mass** |  | **8.06E-06** |
| Unfavorable lifestyle | 1.09 (1.05-1.12) | 2.81E-06 |
| Intermediate lifestyle | 1.19 (1.16-1.21) | 1.39E-36 |
| Favorable lifestyle | 1.24 (1.15-1.32) | 2.78E-06 |
| **Arm fat-free mass** |  | **1.75E-01** |
| Unfavorable lifestyle | 1.09 (1.01-1.17) | 2.99E-02 |
| Intermediate lifestyle | 1.28 (1.22-1.34) | 8.22E-16 |
| Favorable lifestyle | 1.26 (1.08-1.44) | 1.19E-02 |
| **WHR** |  | **1.15E-01** |
| Unfavorable lifestyle | 1.28 (1.22-1.34) | 6.99E-17 |
| Intermediate lifestyle | 1.27 (1.23-1.31) | 5.73E-28 |
| Favorable lifestyle | 1.36 (1.25-1.48) | 2.26E-07 |
| **FFR** |  | **9.35E-06** |
| Unfavorable lifestyle | 1.12 (1.06-1.17) | 7.94E-05 |
| Intermediate lifestyle | 1.25 (1.21-1.29) | 2.48E-26 |
| Favorable lifestyle | 1.26 (1.15-1.38) | 7.22E-05 |

* WHR, waist circumstance/hip circumstance; FFR, whole body fat mass/whole fat-free mass; HR, hazard ratio.

* Values are HR (95%CI) per SD increase obtained from Cox proportional hazard regression model, adjusted for sex, age, assessment center, and Townsend deprivation index.

**Supplementary** **Table 14. Associations of body composition and incident depression after exclusion of the participants with depression symptoms at baseline assessed by PHQ-2**

| **Variable** | **HR (95%CI)** | ***P*** |
| --- | --- | --- |
| Body mass index | 1.19 (1.17-1.22) | 5.21E-56 |
| Body fat percentage | 1.26 (1.23-1.30) | 9.56E-44 |
| Waist circumference | 1.27 (1.24-1.29) | 4.11E-73 |
| Hip circumference | 1.15 (1.13-1.17) | 6.43E-35 |
| Whole body |  |  |
| Whole body fat mass | 1.20 (1.17-1.22) | 1.50E-54 |
| Whole body fat free mass | 1.21 (1.16-1.25) | 2.69E-16 |
| Whole body water mass | 1.21 (1.16-1.25) | 2.15E-16 |
| Trunk |  |  |
| Trunk fat percentage | 1.19 (1.16-1.22) | 8.77E-36 |
| Trunk fat mass | 1.18 (1.16-1.21) | 2.41E-47 |
| Trunk fat free mass | 1.15 (1.11-1.20) | 1.25E-09 |
| Leg |  |  |
| Leg fat percentage | 1.43 (1.38-1.48) | 9.96E-47 |
| Leg fat mass | 1.22 (1.20-1.25) | 6.72E-57 |
| Leg fat-free mass | 1.23 (1.19-1.27) | 4.09E-24 |
| Arm |  |  |
| Arm fat percentage | 1.27 (1.24-1.30) | 6.61E-49 |
| Arm fat mass | 1.18 (1.16-1.20) | 6.74E-59 |
| Arm fat-free mass | 1.21 (1.17-1.26) | 5.26E-17 |
| FFR | 1.25 (1.22-1.28) | 1.78E-48 |
| WHR | 1.31 (1.28-1.34) | 3.81E-64 |

* WHR, waist circumstance/hip circumstance; FFR, whole body fat mass/whole fat-free mass; HR, hazard ratio.

* Values are HR (95%CI) per SD increase obtained from Cox proportional hazard regression model, adjusted for sex, age, ethnicity, assessment center, and Townsend deprivation index.

**Supplementary** **Table 15. Associations of lifestyle component and incident depression after exclusion of the participants with depression symptoms at baseline assessed by PHQ-2**

| **Variable** | **HR (95% CI)** | ***P*** |
| --- | --- | --- |
| Lifestyle score | 0.78 (0.76-0.80) | 8.10E-103 |
| Diet score | 1.03 (0.91-1.17) | 6.63E-01 |
| Sleep score | 0.65 (0.61-0.68) | 9.92E-53 |
| Physical score | 0.84 (0.80-0.88) | 4.66E-12 |
| Sedentary score | 0.75 (0.71-0.79) | 2.47E-26 |
| Social score | 0.79 (0.74-0.84) | 2.29E-12 |
| Smoke score | 0.58 (0.54-0.62) | 6.88E-58 |
| Drink score | 0.88 (0.73-1.05) | 1.66E-01 |

* Values are HR (95%CI) per unit increase obtained from Cox proportional hazard regression model, adjusted for sex, age, assessment center, and Townsend deprivation index. HR, hazard ratio.

**Supplementary** **Table 16. Associations of body composition and lifestyle with incident depression after exclusion of the participants with depression symptoms at baseline assessed by PHQ-2**

| **Variable** | **HR (95%CI)** | ***P*** |
| --- | --- | --- |
| **Body mass index** |  | **3.42E-05** |
| Unfavorable lifestyle | 1.08 (1.04-1.12) | 1.25E-04 |
| Intermediate lifestyle | 1.20 (1.17-1.22) | 1.47E-33 |
| Favorable lifestyle | 1.26 (1.18-1.35) | 3.13E-08 |
| **Body fat percentage** |  | **1.30E-02** |
| Unfavorable lifestyle | 1.11 (1.05-1.17) | 7.84E-04 |
| Intermediate lifestyle | 1.24 (1.20-1.29) | 1.11E-22 |
| Favorable lifestyle | 1.26 (1.15-1.37) | 3.76E-05 |
| **Whole body fat mass** |  | **7.80E-03** |
| Unfavorable lifestyle | 1.08 (1.04-1.12) | 1.27E-04 |
| Intermediate lifestyle | 1.20 (1.17-1.23) | 6.36E-32 |
| Favorable lifestyle | 1.23 (1.15-1.32) | 1.27E-06 |
| **Whole body fat-free mass** |  | **3.98E-01** |
| Unfavorable lifestyle | 1.06 (0.99-1.14) | 1.14E-01 |
| Intermediate lifestyle | 1.25 (1.19-1.31) | 1.41E-13 |
| Favorable lifestyle | 1.26 (1.10-1.43) | 5.30E-03 |
| **Whole body water mass** |  | **3.48E-01** |
| Unfavorable lifestyle | 1.07 (0.99-1.15) | 8.86E-02 |
| Intermediate lifestyle | 1.24 (1.19-1.30) | 1.37E-13 |
| Favorable lifestyle | 1.25 (1.08-1.41) | 8.42E-03 |
| **Trunk fat percentage** |  | **1.30E-02** |
| Unfavorable lifestyle | 1.08 (1.03-1.13) | 2.30E-03 |
| Intermediate lifestyle | 1.17 (1.14-1.27) | 2.81E-18 |
| Favorable lifestyle | 1.17 (1.08-1.26) | 4.50E-04 |
| **Trunk fat mass** |  | **7.80E-03** |
| Unfavorable lifestyle | 1.08 (1.04-1.12) | 3.31E-04 |
| Intermediate lifestyle | 1.18 (1.15-1.21) | 5.70E-27 |
| Favorable lifestyle | 1.20 (1.12-1.29) | 1.98E-05 |
| **Trunk fat-free mass** |  | **2.74E-01** |
| Unfavorable lifestyle | 1.04 (0.96-1.12) | 3.18E-01 |
| Intermediate lifestyle | 1.19 (1.14-1.25) | 5.05E-09 |
| Favorable lifestyle | 1.21 (1.04-1.37) | 2.52E-02 |
| **Leg fat percentage** |  | **3.49E-05** |
| Unfavorable lifestyle | 1.15 (1.07-1.24) | 1.48E-03 |
| Intermediate lifestyle | 1.40 (1.33-1.46) | 1.82E-24 |
| Favorable lifestyle | 1.51 (1.34-1.67) | 1.89E-06 |

| **Supplementary Table 16 (continue)** | | |
| --- | --- | --- |
| **Variable** | **HR (95%CI)** | ***P*** |
| **Leg fat mass** |  | **1.28E-07** |
| Unfavorable lifestyle | 1.09 (1.04-1.13) | 2.38E-04 |
| Intermediate lifestyle | 1.23 (1.20-1.26) | 1.80E-34 |
| Favorable lifestyle | 1.30 (1.21-1.40) | 1.13E-07 |
| **Leg fat-free mass** |  | **7.49E-01** |
| Unfavorable lifestyle | 1.08 (1.01-1.15) | 2.84E-02 |
| Intermediate lifestyle | 1.26 (1.03-1.31) | 1.63E-18 |
| Favorable lifestyle | 1.28 (1.13-1.43) | 1.14E-03 |
| **Arm fat percentage** |  | **2.30E-05** |
| Unfavorable lifestyle | 1.12 (1.06-1.17) | 2.00E-04 |
| Intermediate lifestyle | 1.26 (1.21-1.30) | 1.21E-26 |
| Favorable lifestyle | 1.29 (1.18-1.40) | 3.12E-06 |
| **Arm fat mass** |  | **3.48E-06** |
| Unfavorable lifestyle | 1.08 (1.04-1.12) | 2.45E-05 |
| Intermediate lifestyle | 1.18 (1.15-1.21) | 1.29E-35 |
| Favorable lifestyle | 1.24 (1.16-1.33) | 1.58E-07 |
| **Arm fat-free mass** |  | **3.96E-01** |
| Unfavorable lifestyle | 1.06 (0.99-1.14) | 1.18E-01 |
| Intermediate lifestyle | 1.24 (1.18-1.30) | 4.43E-13 |
| Favorable lifestyle | 1.30 (1.13-1.47) | 1.95E-03 |
| **WHR** |  | **1.81E-01** |
| Unfavorable lifestyle | 1.24 (1.19-1.30) | 2.26E-13 |
| Intermediate lifestyle | 1.26 (1.22-1.30) | 2.54E-27 |
| Favorable lifestyle | 1.32 (1.21-1.43) | 4.45E-07 |
| **FFR** |  | **4.47E-05** |
| Unfavorable lifestyle | 1.10 (1.05-1.16) | 3.64E-04 |
| Intermediate lifestyle | 1.24 (1.20-1.28) | 4.23E-26 |
| Favorable lifestyle | 1.28 (1.17-1.38) | 4.45E-07 |

* WHR, waist circumstance/hip circumstance; FFR, whole body fat mass/whole fat-free mass; HR (hazard ratio)

* Values are HR (95%CI) per SD increase from Cox proportional hazard regression model, adjusted for sex, age, assessment center, and Townsend deprivation index.


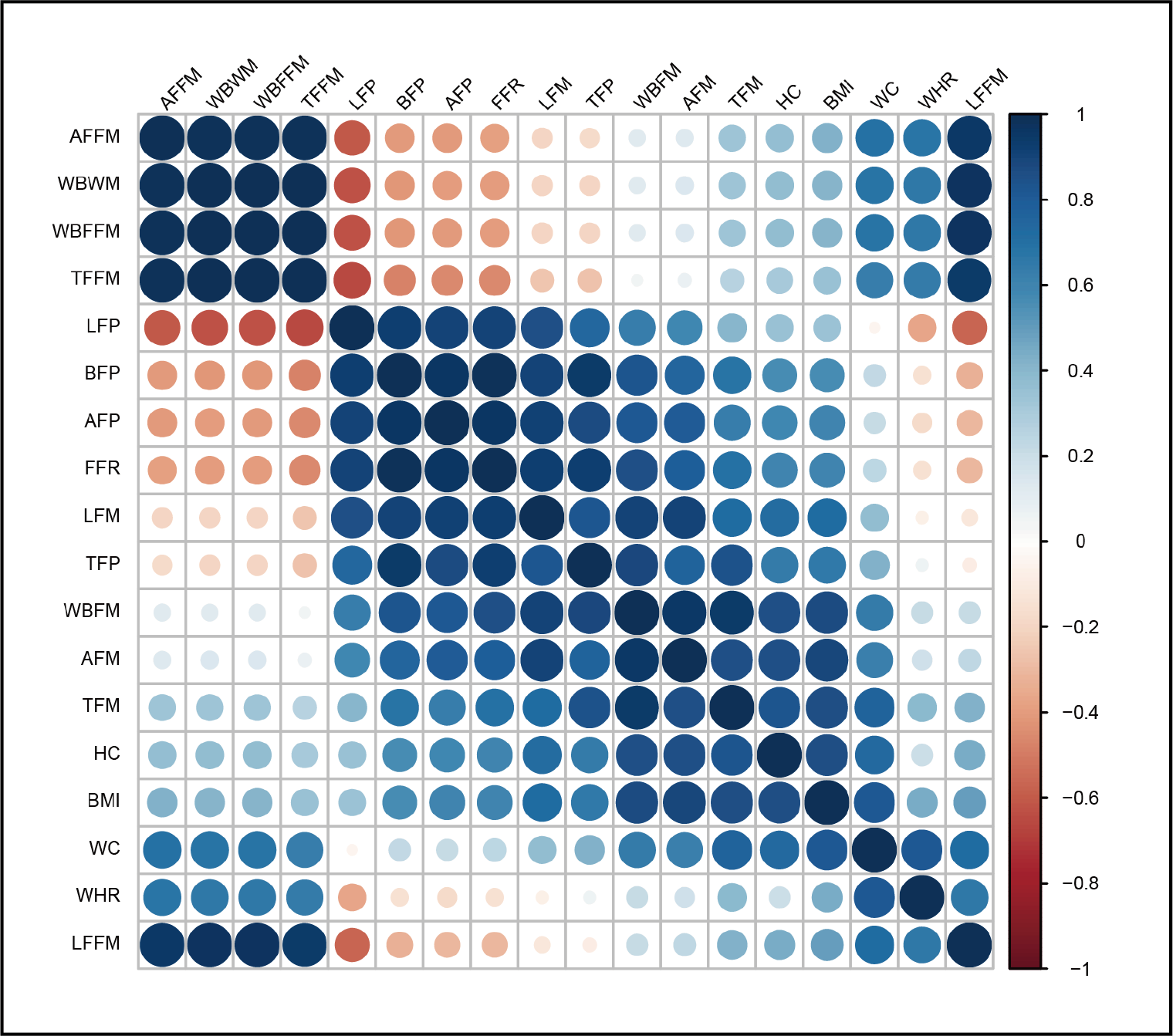


**Supplementary Figure 1 Correlation between body compositions.** The color corresponds to the correlation as the legend describes, and the size represents the *p* value. AFFM, arm fat-free mass; WBWM, whole body water mass; WBFFM, whole body fat-free mass; TFFM, trunk fat-free mass; LFP, leg fat percentage; BFP, body fat percentage; AFP, arm fat percentage; FFR, whole body fat mass/whole fat-free mass; LFM, leg fat mass; TFP, trunk fat percentage; WBFM, whole body fat mass; AFM, arm fat mass; TFM, trunk fat mass; HC, hip circumstance; WC, waist circumstance. WHR, waist circumstance/hip circumstance; LFFM, leg fat-free mass.


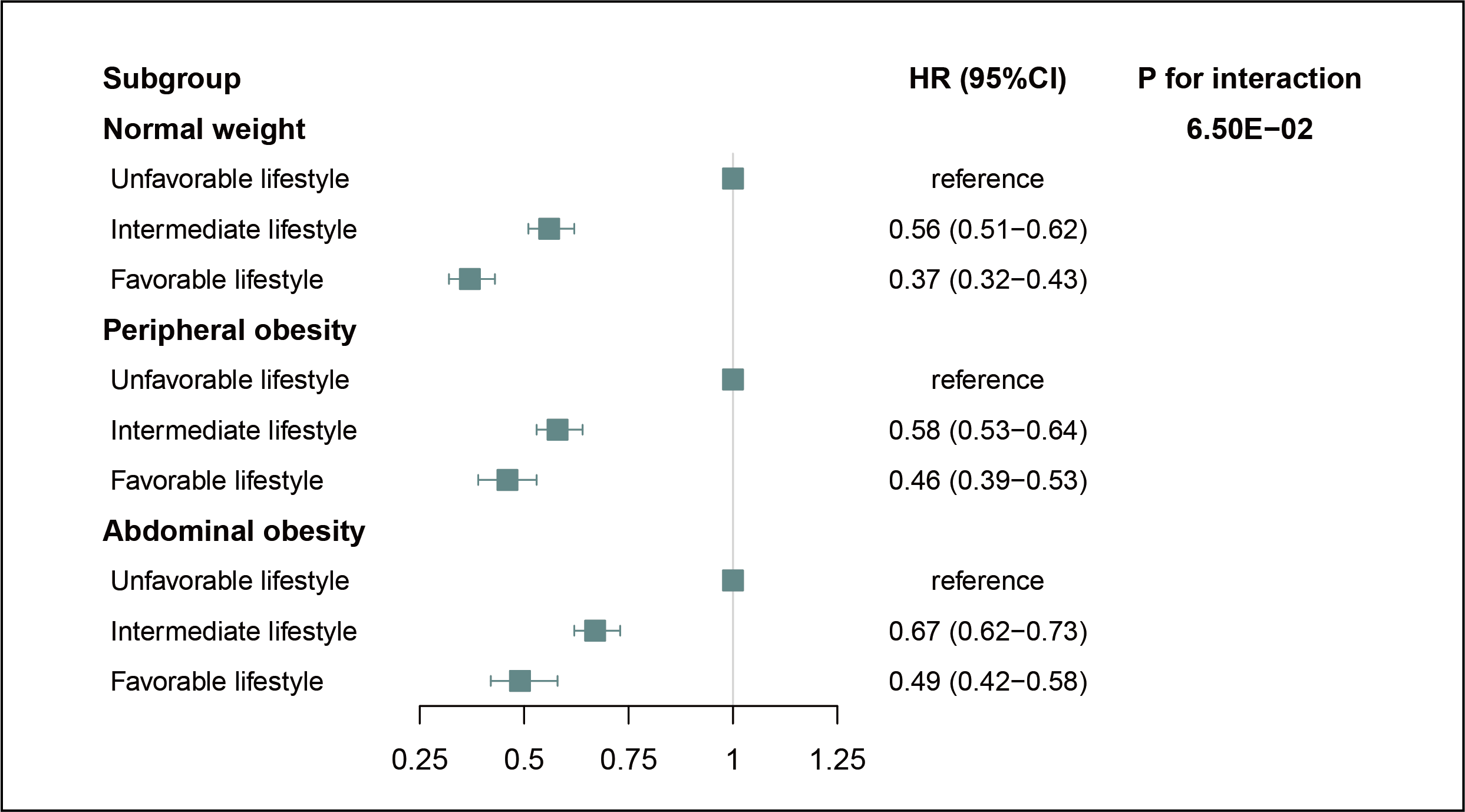


**Supplementary Figure 2 Associations of lifestyle with risks for depression stratified by WHR.** Multivariate-adjusted hazard ratios (HRs) and 95%CIs of depression were estimated from Cox proportional hazards models, adjusted for sex, age, assessment center, and Townsend deprivation index. WHR, waist circumstance/hip circumstance.
